# ANSYS-Based Modeling and Simulation of Electrostatic Oil-Line Sensor

**DOI:** 10.3390/s25154669

**Published:** 2025-07-28

**Authors:** Ruochen Liu, Ge Cai, Jianzhong Sun, Lanchun Zhang

**Affiliations:** 1 School of Automobile and Traffic Engineering, Jiangsu University of Technology, Changzhou 213001, China; liuruochen@jsut.edu.cn (R.L.); zlc@jsut.edu.cn (L.Z.); 2 College of Civil Aviation, Nanjing University of Aeronautics and Astronautics, Nanjing 211106, China; sunjianzhong@nuaa.edu.cn

**Keywords:** electrostatic sensor, oil-line, finite element modeling, simulation analysis

## Abstract

Mechanical components are more difficult to detect at the initial state of failure. To solve this problem, this paper models and simulates the characteristics of an electrostatic oil-line sensor (OLS) wear particles carried in the lubricating oil path are detected. In this study, an OLS that monitors the charge in an oil line using the principle of electrostatic induction is modeled and simulated. The sensor characteristics are simulated and tested using finite element simulation. The sensor efficiency, spatial sensitivity, and length-to-diameter ratio are simulated based on the point charges at different locations. The simulation results show that the sensitivity exhibits different trends when the point charge is inside and outside the probe. The length-to-diameter ratio is proportional to the sensor efficiency, the spatial sensitivity distribution law of multiple charges is consistent with that of a point charge, and the relative deviation rate between the mathematically calculated values and the simulated values is less than 3% under the same conditions. In conclusion, the finite element simulation results of the electrostatic oil line sensor constructed in this study are consistent with the theoretical model calculations and can be used in future mechanical fault diagnosis.

## 1. Introduction

The current vibration monitoring systems face several limitations, including the susceptibility of the site and oil analysis to the environmental and installation space constraints, the complexity of the signal transmission path, and the difficulty of extracting wear particles. Moreover, the acoustic signals are prone to environmental interference and may be insensitive to certain faults and other issues. Therefore, new technologies and methods for the effective online monitoring of the wear states of mechanical systems are urgently required.

During the operation of mechanical equipment, friction due to the relative movement of the contact surfaces produces a series of physical, chemical, and mechanical changes. Numerous friction and wear phenomena occur in the presence of large amounts of static electricity. Electrostatic monitoring in a mechanical system is used to monitor the static charge in the friction and wear regions, as well as to judge the current operating state of the system along with the performance degradation trend with respect to the monitoring signal. In this study, electrostatic oil-line sensors (OLS) based on the principle of electrostatic electricity have been simulated to explore the regularity of change.

Sensor technology has found widespread applications in diverse fields, and researchers have carried out substantial and productive studies utilizing sensors. Yan et al. [1]. constructed the CDTFAFN, an innovative multi-sensor data fusion model, and established a dedicated intelligent mechanical fault diagnosis framework based on this model. Ahuja, P. et al. [2] made significant advances in the study of therapeutic drug glycosylation by employing electrochemical biosensors. Wang et al. [3] used a grating sensor to measure blood pressure and heart rate, obtaining pulse waveforms equivalent to vascular pulsations. In real life, electrostatic sensors are primarily used in various applications such as pneumatic solid flow measurement, measurement of particulate emissions, fluidized bed monitoring, online particle size measurement, burner flame monitoring, measurement of mechanical system speed and radial vibration, monitoring of conveyor belt, mechanical wear, and human activity, etc. Electrostatic sensors mainly monitor the aero-engine states and bearing wear sites when used in the mechanical wear states [4]. Zhang [5] and Qian et al. [6] used multiple electrode arrays to study the characteristics of gas–solid two-phase flow in a square pipe and measured the cross-sectional velocity and concentration distribution of particles under thin conditions of different wind speeds and particle mass flow rates. Both authors adopted mathematical models that were rather complicated. Zhang and Feng et al. [7,8] used electrostatic sensors to study the aero-engine gas path. The difference between the works of Zhang and Feng was that Zhang made sensors and designed circuits to eliminate noise and interference in electrostatic signals, while Feng optimally designed sensors according to their characteristic parameters. Zhong et al. [9] used electrostatic sensors to obtain accurate information about charged particles, such as the particle centroid and particle space position. Ali et al. [10] used electrostatic sensors based on multi-physical field coupling to study particle motion trajectories and complex gas path geometries. These studies have demonstrated the use of electrostatic sensors in various applications.

The above researchers used electrostatic sensors to study different aspects. Several authors have used different shapes of electrostatic sensors in their research. Liu [11,12] presented a three-dimensional (3D) model of two sensors for electrostatic monitoring. Mao [13] proposed and tested the mathematical model of an OLS using a test bed. Heydarianasl [14] established mathematical models and theoretically analyzed various shapes of electrostatic sensors, such as rings, quarter rings, and rectangles. Yu [15] developed a 3D finite element model to analyze the coupled interactions of electrostatic forces, fluid–solid dynamics, and multiple entanglements in a comb resonance electrostatic sensor. Heydarianasl and Hu et al. [16,17] used particle swarm optimization and preamplifier optimization methods, respectively, to optimize electrostatic sensors with different shapes, which improved the overall sensitivity of the sensors. Most of the above studies on electrostatic sensors were based on mathematical models; the use of 3D simulation software was limited, and research on the performance parameters of the sensor itself was not comprehensive. So, more and more people are now choosing to solve problems with finite elements. Wang et al. [18] simulated the process of fatigue crack extension by using a combination of methods with finite elements. Solhmirzaei et al. [19] used the finite element method to test the beams. Currently, most of the studies on OLS mainly focus on the quantity, shape and its applications, while the exploration of its intrinsic properties is still relatively insufficient. For this reason, this paper adopts the finite element modeling simulation method to analyze the sensitivity and efficiency characteristics of OLS in depth in order to fill this research gap.

In this study, the process of charge sensing by OLS is modeled by imposing boundary condition constraints, creating a simulation environment, and applying a charge excitation lee. The change in sensor sensitivity is observed by varying the position of the point charge and the diameter, length, and H/D ratio of the probe. In real life, the charges generated by faulty components are not only point charges, so the study of sensitivity distribution under multiple charges is also necessary. Finally, by comparing the distribution of sensitivity in the axial radial direction between the mathematical model and the simulation model for the same diameter and length of the probe, the correctness of the simulation model can be determined. The correct modeling simulation in this paper reduces the complexity of the mathematical model and describes, in detail, issues such as the effect of the charge position on the sensor parameters. This provides a reference for subsequent studies of multi-sensor and other dynamic sensor characteristics.

## 2. Finite Element Model

An oil line electrostatic sensor detects charged particles based on the principle of electrostatic monitoring. The necessary modeling of the oil line sensor in ANSYS software 19.2 provides a basis for the simulation work. The details of the specific 3D modeling and material selection are explained in the following sections.

### 2.1. Principle of Electrostatic Sensing

**Figure 1:**
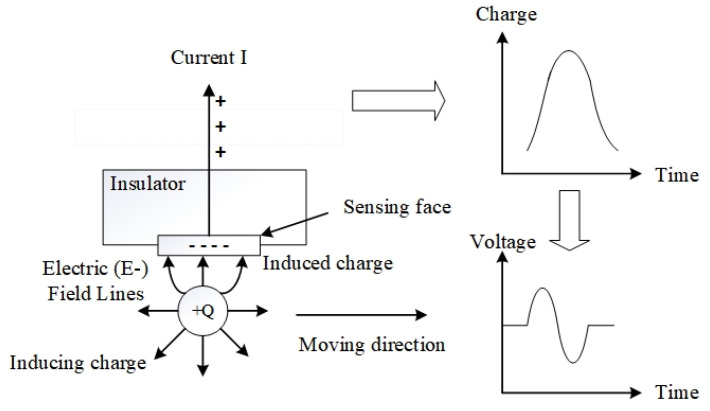
Schematic of the OLS monitoring principle.

Oil line electrostatic sensors are typically installed in mechanical systems with lubrication circuits. Liu et al. [20] clarified the principles of electrostatic monitoring. Oil line sensors are mainly sensitive to charged abrasive particles in the main circuit, and the lines of the electric field concentrate on the probe-sensing area of the sensor when charged particles in the lubricating fluid pass through it. The proximal end of the probe generates a potential opposite to that of the detected charge, while the more distant end of the probe surface maintains a charge with the same potential as the detected charge. The wear state of the system is determined by monitoring the charge information of the charged abrasive particles, while the friction charge generated between the liquid flowing in the pipe and pipe wall also contributes to the sensor monitoring signal. Figure 1 presents a schematic of the OLS monitoring principle, illustrating the electrostatic monitoring mechanism of the oil line electrostatic sensor.

**Figure 2:**
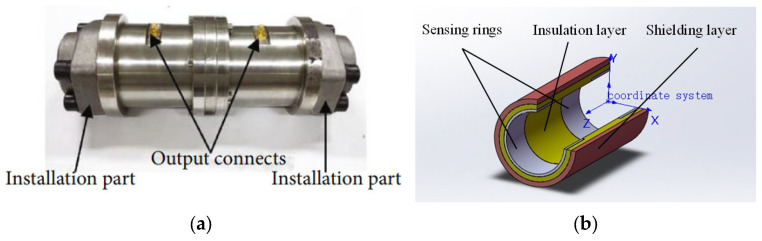
Sensors: (**a**) physical drawing; (**b**) 3D model.

A physical diagram (the sensor was developed by our research team and produced in an industrial facility) and a 3D model of the commonly used oil line sensor are presented in Figure 2. Typically, a sensor consists of a shielding layer, a sensing layer, and an insulating layer. The internal sensing surface and insulation layer are enclosed, leaving only the shielding layer and the microcontactor visible from the outside. The two ends of the sensor are connected to the oil pipe through a standard-size flange, allowing the oil to flow through. The microcontactor is used to output the monitored electrostatic signal. The inner diameter of the sensor is usually the same as that of the connected pipe to avoid affecting the circulation circuit.

### 2.2. Discretization of Electrostatic Sensor

**Figure 3:**
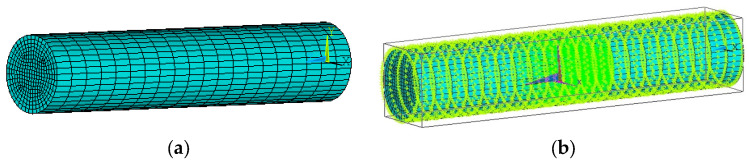
Discretization of a sensor in ANSYS for finite element analysis: (**a**) meshing; (**b**) stimulus application.

The 3D electrostatic field finite element method has been combined with the structure size and shape of OLS to establish the OLS model. The model uses a solid 122 unit type, with the pipe length set to 100 mm. Both the inner diameter of the channel and the diameter of the sensing electrode are set to 20 mm, while the initial length of the induction electrode is set to 10 mm. To meet the high precision requirements, the sensor needs to be refined; thus, the channel unit is divided into 1 mm segments using a mapping grid. Considering the actual application of the sensor, grounding the shield layer and the channel is necessary to prevent the external electric fields from affecting the electrostatic signal induced on the sensing surface. Therefore, a 0 V potential is applied to the shield layer, the boundary conditions of the model are set, and the sensor is an equipotential body. Additionally, since the probe induction surface is an equipotential surface, the nodes on the induction surface must be coupled to ensure they maintain the same potential. In the electric field analysis, the charged particles are considered as point charges, which can be simulated by applying a point charge to the grid nodes inside the pipe. For the convenience of the calculation, and to prevent the low sensitivity from leading to large errors, the charge of the applied point charge is 1 C. The specific sensor modeling grid division and excitation application diagrams are shown in Figure 3.

We consider the electrode and the surrounding medium as a complete electrostatic system, with the medium in the pipe being air. Due to the phenomenon of electrostatic induction, when a charge passes through the probe electrode, an equal amount of opposite sign-induced charge is generated on the surface of the probe electrode, and the amount of charge on the inner and outer surfaces of the electrode varies according to the position of the charge relative to the ring electrode. Therefore, the interaction between the charge and the electrode satisfies the following boundary conditions:

(1) $$\begin{array}{c} \left\{\begin{array}{c} \nabla \cdot \left(\nabla \varphi \left(x,y,z\right)\right)=-\frac{\rho \left(x,y,z\right)}{\varepsilon_{0}}\\ \varphi \left(\Gamma_{p}\right)=0\cup \varphi \left(\Gamma_{i}\right)=0\cup \varphi \left(\Gamma_{t}\right)=0\\ \sigma \left(x,y,z\right)=\varepsilon_{0}\vec{E}\left(x,y,z\right)=-\varepsilon_{0}\nabla \varphi \left(x,y,z\right) \end{array}\right. \end{array}$$

where $\varphi \left(x,y,z\right)$ is the potential distribution; $\varepsilon_{0}$ is the relative dielectric constant; $\rho \left(x,y,z\right)$ is the charge density distribution; $\Gamma_{p}$, $\Gamma_{i}$, and $\Gamma_{t}$ are the boundaries of the probe, insulator, and shield layer, respectively; $\sigma \left(x,y,z\right)$ is the charge density of probe induction; and $\vec{E}\left(x,y,z\right)$ is the electric field strength.

In the Gaussian theorem and electrostatic field theory, the induced charge Q is calculated as follows:

(2) $$\begin{array}{c} Q=\int_{s}D\left(x,y,z\right)dS=\varepsilon_{0}\int_{s}E\left(x,y,z\right)dS \end{array}$$

where *S* is the surface area of the sensor; *D*(*x*, *y*, *z*) is the magnetic flux distribution; and dS is the area element.

Since the simulation model in this study is not complex, the induced potential of the induced surface due to a point charge can be solved using the wavefront solver. However, the primarily objective is to determine the amount of charge induced on the probe induction surface by a point charge, which depends only on the strength of the electric field perpendicular to the induced surface. Therefore, to calculate the induced charge, first, the electric field intensity vertically induced surface should be extracted and then used to compute the induced charge on the probe.

### 2.3. Material Models for Electrostatic Sensor

The sensing surface of the sensor is used for determining the electric charge in the wear area, generally using copper, and the insulation layer is used to ensure the reliability of the induced charge quantity and to prevent the leakage of the induced charge, generally using polytetrafluoroethylene. The shielding layer is mainly used to shield the interference of the external electromagnetic signals with the electrostatic signals, and it is generally made of stainless steel and is grounded. Since the micro contactor and flanges are not involved in electrostatic simulation, they do not require a detailed description. The material selection for each component is listed in Table 1.

## 3. Model Simulation

To express the spatial distribution of the OLS sensing interval more clearly, two performance parameters—spatial sensitivity and sensor efficiency characteristics—have been introduced. The finite element model is used to simulate and analyze the two performance parameters at different axial and radial positions. A comparison is made between the finite element modeling and the mathematical model for both point and multiple charges, leading to more rigorous simulations.

### 3.1. Space Sensitivity Simulation Analysis

Based on the principle of electrostatics, the generated electrostatic field varies depending on the position of point charge within the pipe, leading to changes in the amount of charge induced by the electrostatic sensor. To clearly illustrate how the induced charge in the OLS varies with the spatial position of the point charge, spatial sensitivity is introduced as a performance parameter. The theoretical formula is as follows:

(3) $$\begin{array}{c} s=\left|\frac{Q}{q}\right| \end{array}$$

where *s* is the spatial sensitivity; *q* is the charge carried by the point charge; and *Q* is the charge that can be induced by the point charge.

In this study, the spatial sensitivity characteristics of OLS mainly focus on the following two aspects: (1) keeping the probe size unchanged and observing the spatial sensitivity distribution by varying the position of the induced charge; (2) analyzing the spatial sensitivity distribution of the induced charge at the same radial position within the pipe while varying the probe size.

**Figure 4:**
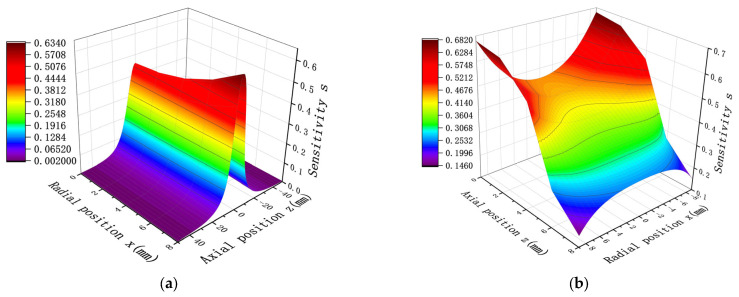
Charge at different radial and axial positions for the probe with D = 20 mm and H = 10 mm: (**a**) sensitivity distribution along the axis at different radial positions; (**b**) sensitivity distribution along the radial direction at different axial positions.

A probe diameter D = 20 mm and length H = 10 mm were set as the initial values, and the sensitivity of the point charge in the sensor at different positions was simulated. Figure 4 presents the findings for this particular distribution. As illustrated in Figure 4a, the radial location is fixed, the sensitivity increases as $\left|z\right|$ decreases, and the sensitivity reaches a maximum at z = 0; i.e., the sensitivity is maximum when the point charge is located on the center of the cross-section of the probe. As shown in Figure 4b, the axial position is fixed. When $\left|z\right|$ < 5; i.e., when the point charge is located inside the probe, the sensitivity decreases with a decrease in $\left|z\right|$. As the point charge approaches the central axis of the probe, the sensitivity decreases and reaches a minimum at the central axis x = 0. Furthermore, when $\left|z\right|$ > 5; i.e., when the point charge is located outside the probe, the sensitivity increases with a decrease in $\left|x\right|$. Therefore, the shorter the distance of the point charge from the central axis of the probe, the greater the sensitivity, and the maximum is reached at the central axis x = 0.

**Figure 5:**
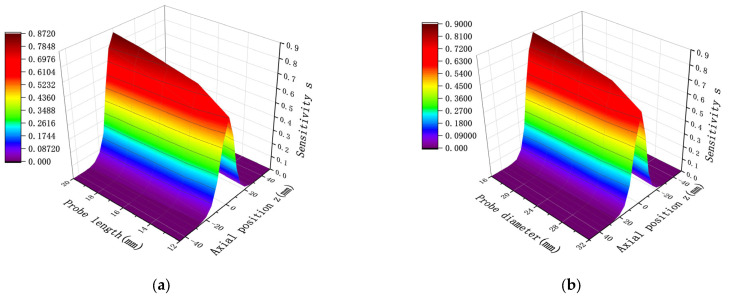
Distribution of point charge sensitivity in the axial direction at the same radial position with varying probe size: (**a**) probe diameter D = 20 mm, while length varies; (**b**) probe length H = 20 mm, while diameter varies.

The study of spatial sensitivity with respect to changes in probe size primarily aims to observe the effect of varying one parameter of spatial sensitivity while keeping either the probe length or the diameter fixed. The sensor probe length and diameter were fixed, and a spatial sensitivity simulation curve was obtained for a point charge located at the same radial location, with x = 0. The distribution results are shown in Figure 5. As demonstrated in Figure 5a, when the radial position is fixed, an increase in the probe length of the sensor results in higher sensitivity, following an overall trend of increasing sensitivity with length. Additionally for sensors with the same probe length, sensitivity increases as the position approaches x = 0. Therefore, when the probe diameter is fixed, increasing the probe length is an effective way to enhance sensitivity. As evident in Figure 5b, the sensitivity distribution can be divided into two regions. When the point charge is located outside the corresponding length of the probe, an increase in diameter leads to higher sensitivity. However, when the point charge is within the corresponding length of the probe, an increase in diameter reduces the sensitivity. Since sensor research primarily focuses on improving sensitivity within the inner region of the probe, reducing the sensor diameter while keeping the probe length fixed is also an effective approach to enhance sensitivity.

### 3.2. Comparative Analysis of Finite Element and Mathematical Model

**Figure 6:**
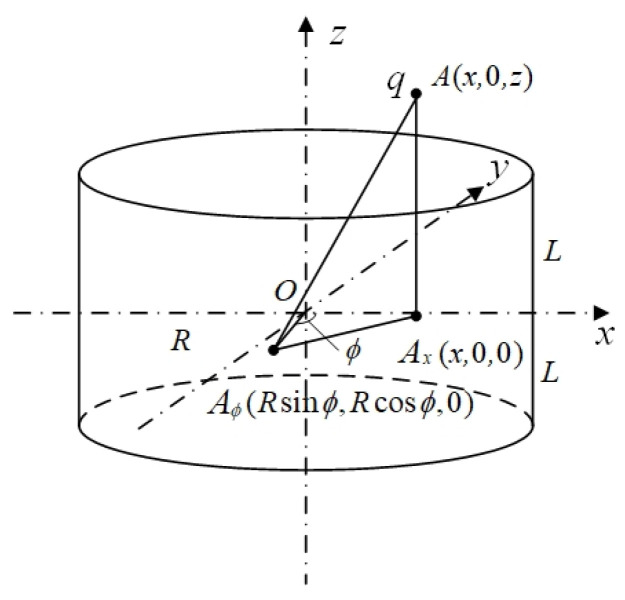
Mathematical modeling of oil line electrostatic sensor with point charge.

In this paper, we will use the method of comparing with the mathematical model to verify the correctness of the constructed finite element simulation model in order to increase the credibility of the model. In the mathematical model, to obtain the charge Q induced by the ring probe, establishing a theoretical model with the center O of the ring probe as the origin, the axis of the probe as the *z*-axis, and the middle cross-section of the probe as the xOy plane is necessary, as shown in Figure 6.

The probe has a length of 2 L and a diameter of 2 R. Suppose a point charge q is located at point A (x,0,z) inside the probe. The projection of point A onto the plane is denoted as $A_{x}$, where x represents the radial position of the point charge within the probe, and z represents the axial position of the point charge within the probe. At this point, the induced charge Q on the probe is given by the following:

$$Q=\frac{Rq}{2\pi }\int_{0}^{\pi }\frac{R-xcos\emptyset }{R^{2}+x^{2}-2Rxcos\emptyset }\cdot \left[\frac{z+L}{\sqrt{{\left(z+L\right)}^{2}+R^{2}+x^{2}-2Rxcos\emptyset }}-\frac{z-L}{\sqrt{{\left(z-L\right)}^{2}+R^{2}+x^{2}-2Rxcos\emptyset }}\right]d\emptyset$$

(4) $$\left(-R<X<R\right)$$

where ∅ is the angle between the probe on the plane and the line between $A_{\emptyset }$ and the coordinate origin and O$A_{x}$.

**Figure 7:**
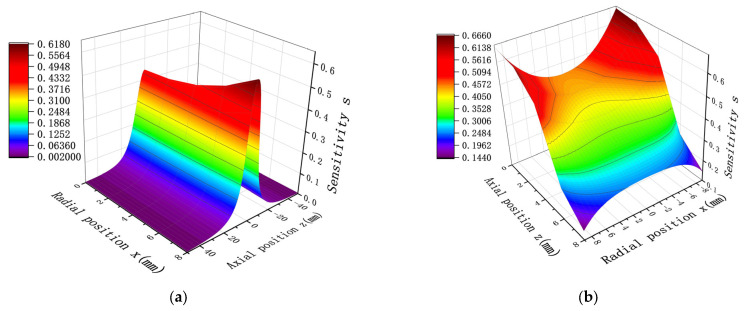
Charge at different radial and axial positions for the probe with D = 20 mm and H = 10 mm under mathematical modeling: (**a**) sensitivity distribution along the axis at different radial positions; (**b**) sensitivity distribution along the radial direction at different axial positions.

In order to verify the reasonableness of the simulation model, space sensitivity is selected as the research parameter in this study, and with the typical dimensions of the electrode diameter and height set to D = 20 mm and H = 10 mm, respectively, the induced charge of the probe electrode at different radial and axial positions is calculated by establishing a mathematical model, and the results are compared and analyzed with the simulation data shown in Figure 4. Under the above conditions, the mathematical modeling calculations obtained are shown in Figure 7.

By comparing the results of Figure 4 with those of Figure 7, it can be found that under the same values of D and H, the mathematical model calculation results of spatial sensitivity show distribution trend that is a highly consistent with the simulation results. Specifically, when the radial position is fixed, the sensitivity achieves the maximum value at z = 0. When the axial position is fixed, in the range of $\left|z\right|$ < 5 mm, the sensitivity decreases with the decrease in $\left|z\right|$; while in the region of $\left|z\right|$ > 5 mm, the sensitivity increases with the decrease in $\left|x\right|$. After quantitative analysis, the relative deviation rates (based on the average value) between the mathematical model and the simulation model in the radial and axial directions are 2.71% and 2.36%, respectively.

**Figure 8:**
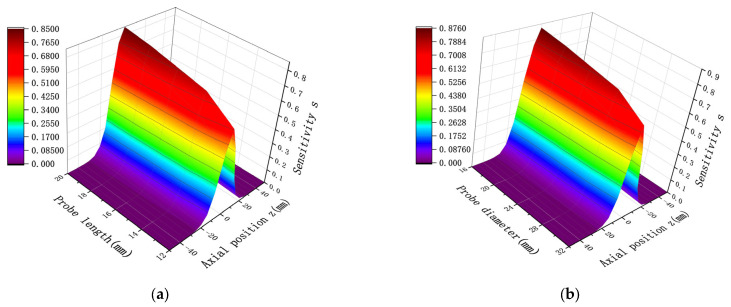
Distribution of point charge sensitivity in the axial direction at the same radial position under the mathematical model with different probe dimensions: (**a**) probe diameter D = 20 mm, with different lengths; (**b**) probe length H = 20 mm, with different diameters in two dimensions.

In the following, we choose the same diameter and length conditions as in Figure 5, resulting in the mathematical calculations shown in Figure 8.

Comparison of the results in Figure 5 and Figure 8 shows that the results of the distribution trend of spatial sensitivity remain the same for the same D and H. Specifically, as the sensor probe length H increases and the probe diameter D decreases, the spatial sensitivity increases, and the sensitivity still takes the maximum value at Z = 0. After quantitative analysis, the relative deviation rates (based on average values) between the mathematical model and the simulation model for length and diameter are 2.52% and 2.67%, respectively. This study establishes a validated simulation model for predictive fault monitoring in rotating machinery systems. This small deviation range fully verifies the accuracy and reliability of the established mathematical model. There may be two reasons for the relative deviation rate between the mathematical model and the simulation model: first, the mathematical model only considers the positional reason when calculating, while the modeling needs to consider the material and other issues; second, when the distance between the charged particles and the induction surface of the detector pole is closer, the center area of the sensor has higher sensitivity to the monitoring of the particles, has the ability to capture the particles near the center, and attracts the electric field lines more strongly, so the simulation results will be slightly higher than the mathematical calculation results. This reduces the number of assumptions required, as well as the cumbersome formulas and steps involved in mathematical calculations, providing a more convenient way of calculating.

### 3.3. Sensor Efficiency Simulation Analysis

Sensor efficiency is divided into theoretical and working efficiencies. The theoretical efficiency can be obtained through simulation calculations, whereas the working efficiency must be obtained experimentally. Therefore, this study focuses on analyzing the theoretical efficiency based on the simulation results. The theoretical efficiency is expressed as follows:

(5) $$\begin{array}{c} \eta =-\frac{Q_{c}}{q}\times 100\% \end{array}$$

where η is the theoretical efficiency of the sensor; and $Q_{c}$ is the amount of electricity induced by the probe when the point charge is at C.

The theoretical efficiency of the sensor calculated using Equation (5) can also be considered as the spatial sensitivity at the particular location.

According to the spatial sensitivity distribution law caused by changes in the probe size, discussed in Section 3.1., an important parameter influencing the spatial sensitivity of the probe is the length-to-diameter ratio. To achieve higher sensitivity, this ratio must be increased. Therefore, to analyze the theoretical efficiency of the sensor more directly, we assess efficiency based on the value of λ.

(6) $$\lambda =\frac{H}{D}$$

where λ is length-to-diameter ratio; *H* is the length of the probe; and *D* is the diameter of the probe.

Since measuring points within the sensor probe area are diverse and can be distributed anywhere, fixing the measuring points is necessary to study the relationship between λ and sensor efficiency. Considering the above analysis of the spatial sensitivity of the sensor, the sensitivity change at the center of the sensor probe (Point O) is the most significant. Therefore, point O can be used as a reference point to evaluate the efficiency of the sensor.

**Figure 9:**
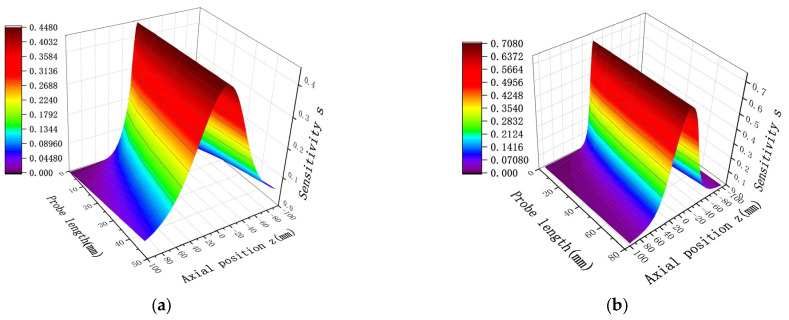
Sensitivity distribution of point charge in the axial direction with varying H and constant λ: (**a**) λ = 0.5; (**b**) λ = 1.

λ is fixed and the probe length is changed to observe the sensitivity distribution of the sensor along the axis. The sensitivity distribution for λ = 0.5 and λ = 1 is presented in Figure 9. Clearly, when λ is fixed, regardless of changes in probe length, the sensitivity at point O on the central axis remains constant and clustered at a single value. However, when the λ value changes, the sensitivity at point O also changes, indicating a variation in sensor efficiency. The same conclusion holds when λ is fixed and the probe diameter is varied.

**Figure 10:**
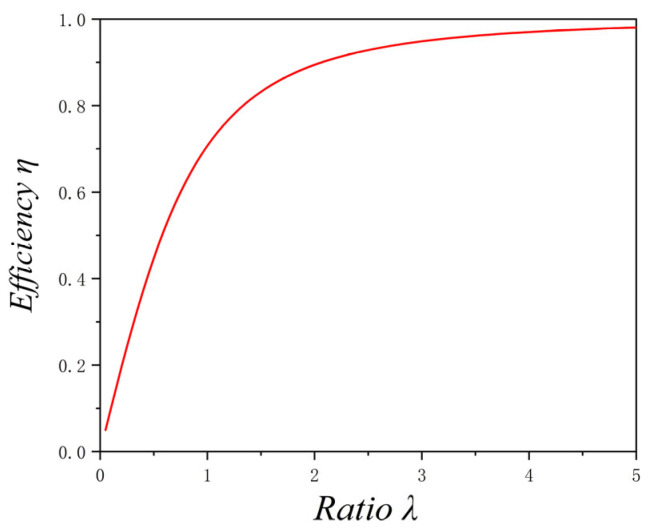
Variation of sensor efficiency at point O with λ.

To verify the law, the simulated sensitivity variation trend (variation of the theoretical efficiency of the sensor) under different values of λ is shown in Figure 10. As illustrated in Figure 10, the sensor efficiency increases as λ increases. However, the efficiency rises rapidly at first, and when λ reaches 3, the increase begins to level off. Additionally, the theoretical efficiency remains consistently below 1.

### 3.4. Comparative Analysis of Point and Multiple Charges

In reality, when monitoring component failure using an electrostatic sensor, the particles produced in the wear area are not point-charge particles, but rather multiple charge particles, with their number constantly changing. According to the superposition principle of the electrostatic field, when multiple charges exist in the induction region, their electric field strengths can be superimposed at any given point in space. Therefore, the output characteristics of multiple applied charges can be simulated based on the results of a single applied charge simulation.

**Figure 11:**
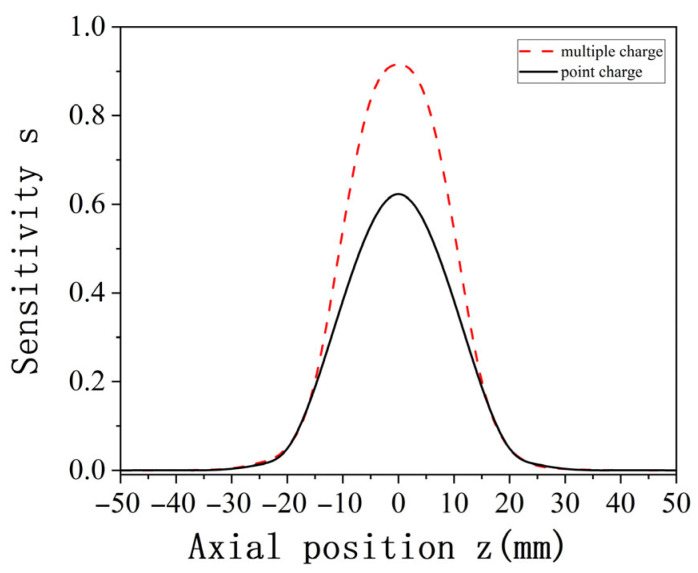
Comparison of point charge and multiple charge spatial sensitivity.

To better investigate the characteristic parameter distribution of the oil line sensors under multiple charge conditions, the approach outlined in Section 2.2 is adopted. The established model, based on the point charge simulation process, is used to simulate and analyze the spatial sensitivity of multiple charges. The simulation results for both point charge and multiple charge spatial sensitivity are shown in Figure 11. Figure 11 demonstrates that the spatial sensitivity distribution trend under multiple charges is consistent with that for a point charge. When the charge is located at the axial position z = 0, corresponding to the central axial section of the pipeline, the sensitivity reaches its maximum. Additionally, the sensitivity distribution on both sides of the radial position z = 0 is symmetric.

**Figure 12:**
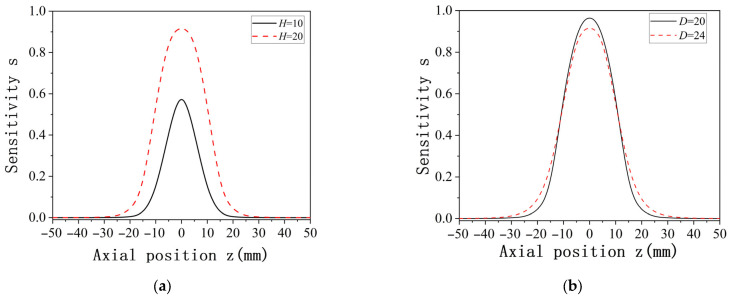
Sensitivity distribution when probe diameter D and length L are changed under multiple charges: (**a**) D = 20 mm, while H is varied; (**b**) H = 20 mm, while D is varied.

The spatial sensitivity under multiple charges also varies with changes in the length and width of the probe. When the probe diameter is fixed while the length is varied, and when the length is fixed while the diameter is varied, the resulting spatial sensitivity distribution under multiple charges is shown in Figure 12. As is evident in Figure 12, despite differences in probe length H and diameter D, the overall sensitivity distribution trend remains unchanged. This trend is consistent with the sensitivity distribution observed under point charge and follows a normal distribution pattern. The sensitivity distribution is symmetric about the axial position z = 0, where the sensitivity reaches its maximum value. Furthermore, in the presence of multiple charges, the sensor sensitivity is proportional to the length of the probe and inversely proportional to its diameter. Higher sensitivity is achieved when the length increases and the diameter decreases. These simulation results confirm that the sensitivity distribution characteristics for multiple charges can be effectively derived from point charge simulation analysis.

## 4. Conclusions

Based on the principle of electrostatic monitoring, the characteristic parameter settings of an oil line electrostatic sensor were studied. This study presents a finite element simulation method using ANSYS software to avoid the complicated calculation of the mathematical models. Considering the influence of different dimensions on the characteristic parameters of the sensor, a finite element analysis was performed. Accordingly, the following conclusions can be drawn from the simulation results:

1. When probe sensitivity increases and the radial position is fixed, the point charge moves closer to the central cross-section of the probe.
2. When the point charge inside the probe moves closer to the central axis while its axial position remains fixed, the sensitivity decreases. At this stage, the sensitivity distribution inside the probe is the opposite of the general sensitivity distribution pattern.
3. The length-to-diameter ratio of the sensor probe is a crucial factor for determining sensor performance. If this ratio changes, efficiency also changes. Generally, an increase in this ratio leads to higher efficiency.
4. The trend of spatial sensitivity distribution under multiple charges is consistent with that observed for point charges.
5. The simulation results of this study were compared with the existing mathematical calculations, and the trends were found to align. Under the same conditions, the relative deviation rates between the mathematically calculated and simulated values of the radial–axial direction are 2.71% and 2.36%, respectively, and the relative deviation rates between the mathematically calculated and simulated values of length and diameter are 2.52% and 2.67%, which are lower than 3%, which verifies the correctness of the finite element model.

Several issues still exist in this study that deserve to be explored in depth and provide a clear direction for subsequent research. These include the following: experimental validation of the OLS simulation model to confirm its accuracy, assess sensor performance, and define input–output relationships; the spatial sensitivity distribution law of moving charges in an electrostatic field and its physical mechanism; the specific causes of the relative deviation between the mathematically calculated values and the simulated values and their optimization methods; and other factors that may affect the measurement accuracy and their compensation mechanisms.

## Figures and Tables

**Table 1:** Oil line electrostatic sensor components and materials, relative dielectric constants, and bulk conductivity.

Components	Materials	Relative Dielectric Constants	Bulk Conductivity/ $(10^{6}s\cdot m^{-1}$)
Shielding layer	Stainless steels	1	2
Insulation layer	Polytetrafluoroethylene	2.1	0
Sensing rings	Copper	1	58
Pipeline	Stainless steels	1	20
Pipeline medium	Air	1	0

## Data Availability

Data are contained within the article.
